# Bladder Cancer Treatment Response Assessment in CT using Radiomics with Deep-Learning

**DOI:** 10.1038/s41598-017-09315-w

**Published:** 2017-08-18

**Authors:** Kenny H. Cha, Lubomir Hadjiiski, Heang-Ping Chan, Alon Z. Weizer, Ajjai Alva, Richard H. Cohan, Elaine M. Caoili, Chintana Paramagul, Ravi K. Samala

**Affiliations:** 1 Department of Radiology, The University of Michigan, Ann Arbor, Michigan 48109 United States; 2 Department of Urology, Comprehensive Cancer Center, The University of Michigan, Ann Arbor, Michigan 48109 United States; 3 Department of Internal Medicine, Hematology-Oncology, The University of Michigan, Ann Arbor, Michigan 48109 United States

## Abstract

Cross-sectional X-ray imaging has become the standard for staging most solid organ malignancies. However, for some malignancies such as urinary bladder cancer, the ability to accurately assess local extent of the disease and understand response to systemic chemotherapy is limited with current imaging approaches. In this study, we explored the feasibility that radiomics-based predictive models using pre- and post-treatment computed tomography (CT) images might be able to distinguish between bladder cancers with and without complete chemotherapy responses. We assessed three unique radiomics-based predictive models, each of which employed different fundamental design principles ranging from a pattern recognition method via deep-learning convolution neural network (DL-CNN), to a more deterministic radiomics feature-based approach and then a bridging method between the two, utilizing a system which extracts radiomics features from the image patterns. Our study indicates that the computerized assessment using radiomics information from the pre- and post-treatment CT of bladder cancer patients has the potential to assist in assessment of treatment response.

## Introduction

Bladder cancer is the fourth most common cancer in men in the United States. The American Cancer Society estimates that bladder cancer will cause 16,870 deaths (12,240 in men, 4,630 in women) in the United States in 2017 with 76,030 new cases being diagnosed (60,490 in men, 18,540 in women)^1^.

Radical cystectomy provides the best local control for patients with localized muscle invasive or recurrent non-muscle invasive bladder cancer. Despite adequate local cancer control, approximately 50% of patients who have undergone cystectomy develop metastases within two years of cystectomy and subsequently die of the disease. This is likely due to the presence of regional or distant microscopic metastatic disease at the time of surgery.

Neoadjuvant chemotherapy prior to cystectomy utilizing a cisplatin-based regimen has been shown to decrease the probability of finding extravesical disease when compared to radical cystectomy alone and also improve survival^2–4^. Patients with a complete local response within their bladder following neoadjuvant chemotherapy (approximately 30% of the patients) have 5-year recurrence free survival, equivalent to patients who undergo cystectomy for non-muscle invasive disease (85–90%). Currently, there is no reliable method for predicting the response of an individual case to neoadjuvant chemotherapy prior to or during its administration. As a result, some patients may suffer from adverse reactions to the drugs without achieving beneficial effects. Furthermore, these patients may miss the opportunity to receive alternative therapy as a result of the deterioration of their physical condition from the initial chemotherapeutic regimen.

Early assessment of therapeutic efficacy and prediction of treatment failure would help clinicians decide whether to discontinue chemotherapy at an early phase before additional toxicity develops, thus improve the quality of life of a patient and reduce unnecessary morbidity and cost. The ultimate goal is to improve survival for those with a high risk of recurrence while minimizing toxicity to those who will have minimal benefit. Therefore, development of an accurate and early predictive model of the effectiveness of neoadjuvant chemotherapy is important for patients with bladder cancer. Many bladder cancers are well visualized with CT^5–7^. It might be possible to characterize the responsiveness of a cancer to chemotherapy by changes in its imaging characteristics.

Radiomics is the study of using radiological images to analyze anatomic or physiologic abnormalities based upon the imaging characteristics^8–10^. Previous studies decoded tumor phenotypes^11^ using radiomics features, or evaluated the correlation between radiomics features with radiation therapy dose and radiation pneumonitis^12^ in lung CT. A review paper on radiomics for lung cancer concluded that radiomics have the potential to improve lung cancer diagnosis^13^.

One promising method that may be useful for extracting image information for radiomics application is convolution neural network (CNN). A CNN learns the underlying patterns and features in the input training images using many small convolution kernels, which contain the weights that the network incorporates the knowledge learned from the training images and generalizes it to recognize unknown images outside the training set. CNN has been used for many years and has been implemented successfully to classify different types of medical image patterns^14–22^. With the recent increase in the power for parallel computing, primarily resulting from the development of powerful graphics processor units (GPUs), training of a very large CNN with many layers and connections in the network has become feasible. Deep-learning convolutional neural networks (DL-CNN) are CNNs capable of learning complex patterns using large numbers of input images via a large neural network with many connections. The application of DL-CNN to various tasks in medical image analysis has been examined recently^23–26^.

In this study, we explored the possibility that radiomics-based predictive models might be able to distinguish between bladder cancers that have fully responded to chemotherapy and those that have not, based upon analysis of pre- and post-treatment CT images. We evaluated three unique radiomics predictive models, which employ different fundamental design principles: (1) a pattern recognition method (DL-CNN), (2) a more deterministic radiomics feature based approach (RF-SL), and (3) a bridging method between the two, which extracts radiomics features from image patterns (RF-ROI). We studied both the properties of the different predictive models and the relationship between these different radiomics approaches. We also compared the performance of the models in predicting a complete response of bladder cancer to neoadjuvant chemotherapy with that of expert physicians.

With the approval of the Institutional Review Board (IRB), a training data set of 82 patients with 87 bladder cancers who were evaluated with CT before and after the administration of neoadjuvant chemotherapy was collected retrospectively. An additional 41 patients with 43 cancers were collected as a test set. Chemotherapy regimens used for the majority of these patients were MVAC (methotrexate, vinblastine, doxorubicin, and cisplatin treatment), while other patients were treated with regimens including carboplatin, paclitaxel, gemcitabine, and etoposide. The pre-treatment scans were acquired approximately 1 month (max 3 months) before the first cycle of chemotherapy. The post-treatment images were acquired after the completion of three cycles of chemotherapy, generally within 1 month (max 2 months) of cessation of the therapy. The pre-post-treatment scans were on average acquired 4 months apart. Each patient underwent cystectomy at the end of his or her chemotherapy, usually 4–6 weeks after completion of neoadjuvant chemotherapy, which is generally within 4 months after the post-treatment CT scan. Pathology obtained from the bladder at the time of surgery was used to determine the final cancer stage after chemotherapy and was used as the reference standard to determine whether or not the patient had responded completely to treatment.

### DL-CNN predictive model

**Figure 1:**
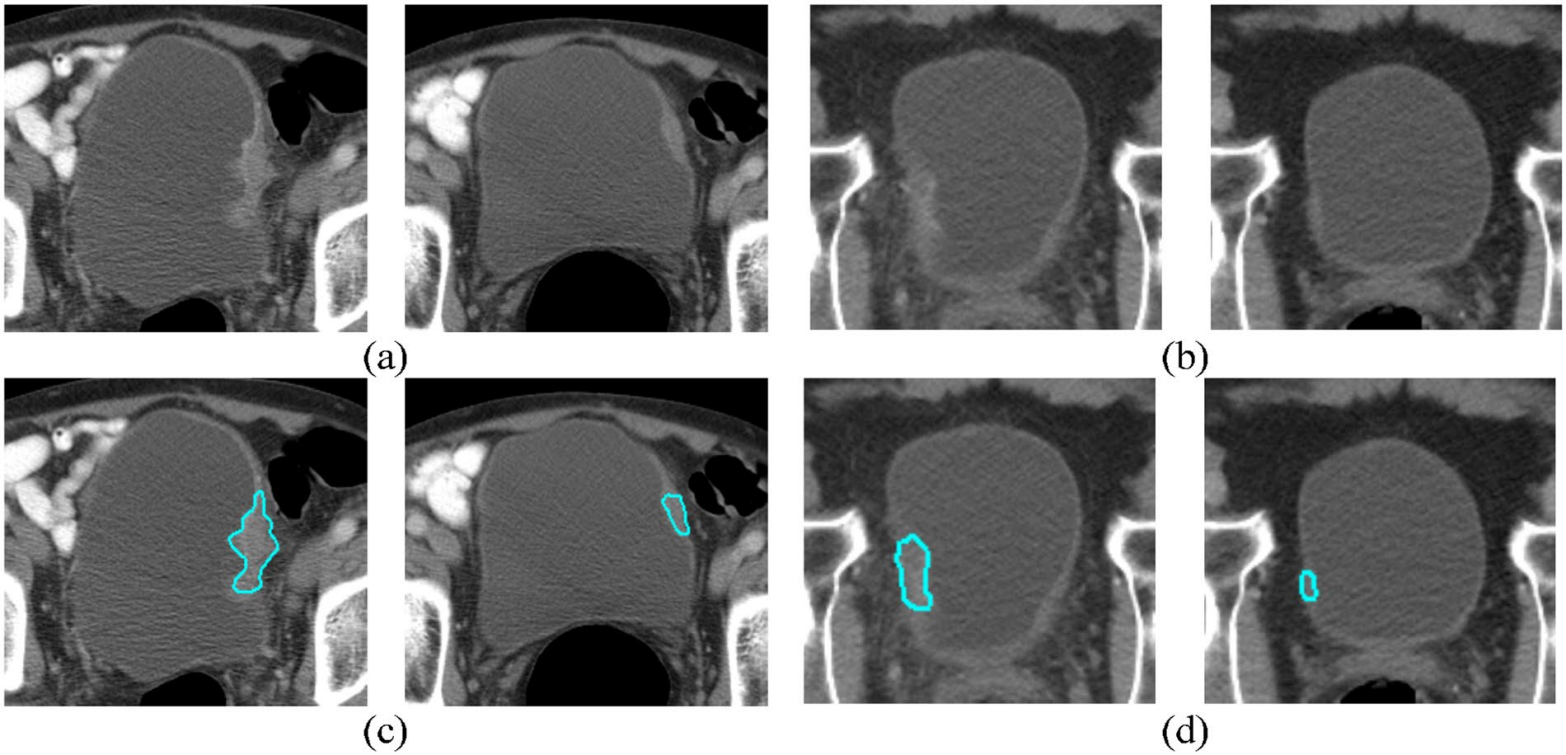
Bladder lesion segmentations. Two segmented bladder cancers are illustrated. The lesions in the pre- and post-treatment scan pairs shown in (**a**,**b**) are segmented using AI-CALS, as shown in (**c**,**d**), respectively. The pre-treatment scan is on the left and the post-treatment scan is located on the right of each pair.

**Figure 2:**
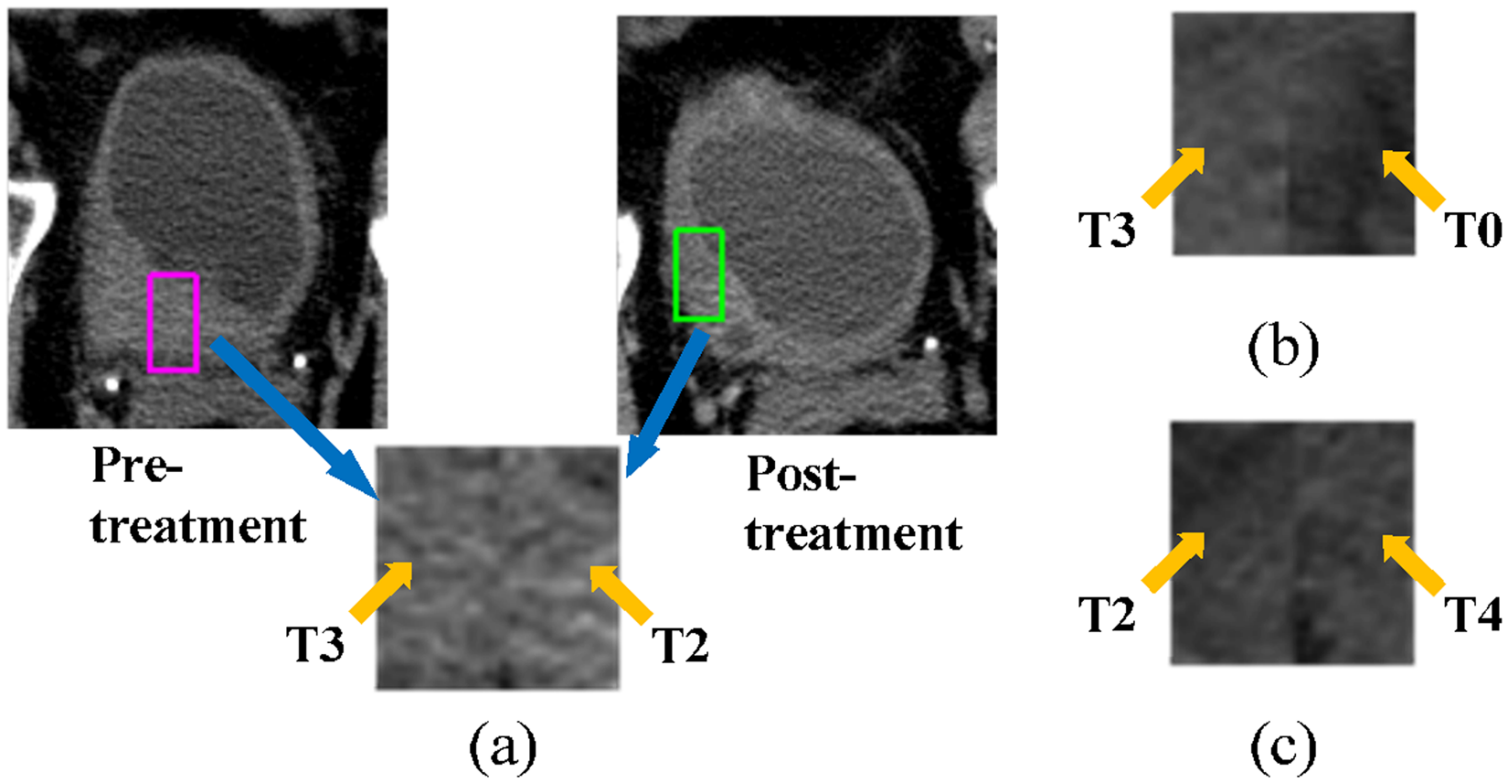
Creating ROIs to train the DL-CNN. (**a**) ROIs were generated by combining regions from the pre- and post-treatment scan lesions. In this example, the pre-treatment stage was T3, and the post-treatment stage was T2. Therefore, the ROI was labeled as being greater than stage T0 after treatment. (**b**) ROI of a case that was stage T3 pre-treatment and stage T0 after treatment. (**c**) ROI of a case that was stage T2 pre-treatment and stage T4 post-treatment. Therefore the ROI was labeled as greater than stage T0 after treatment.

**Figure 3:**
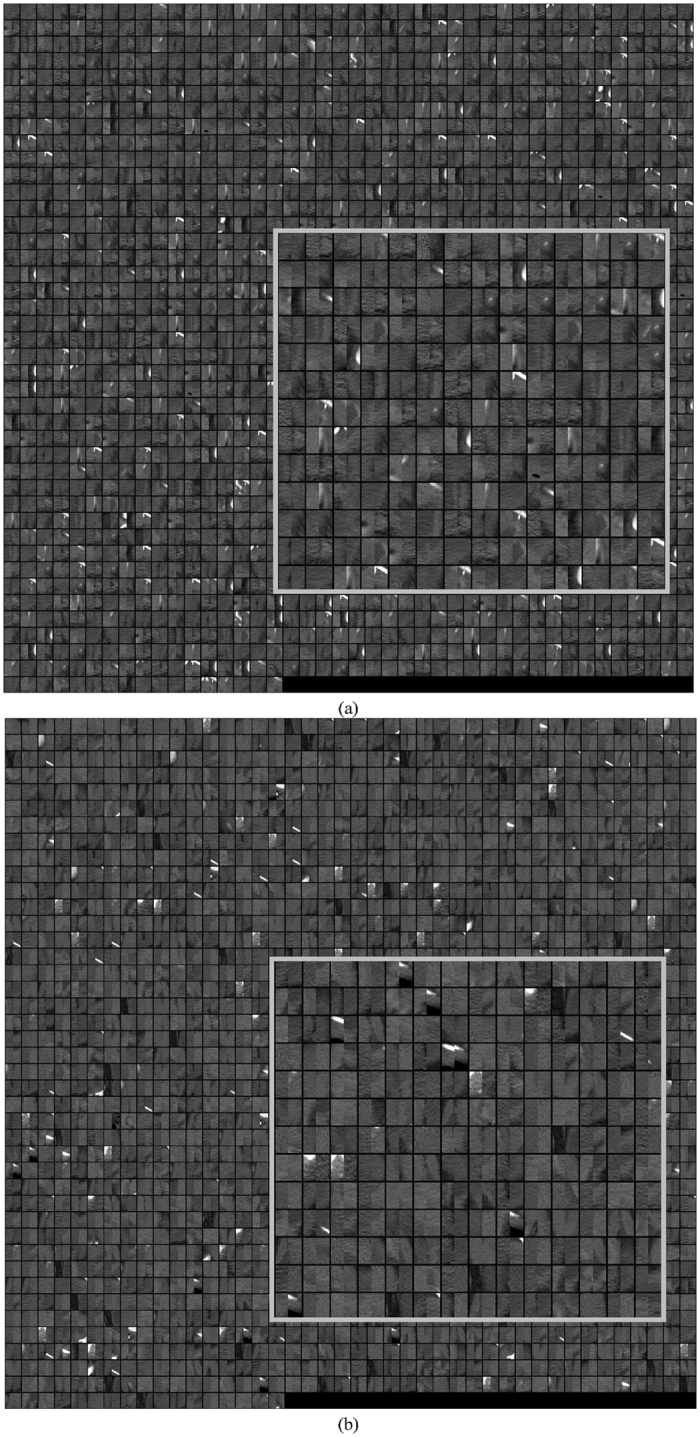
Subset of Paired ROIs used to train the DL-CNN. Each ROI is 32 × 32 pixels. (**a**) ROIs that were labeled as being stage T0 after treatment. (**b**) ROIs that were labeled as being greater than stage T0 after treatment. A portion of ROIs in each class is zoomed in to illustrate the content of typical ROIs.

**Figure 4:**
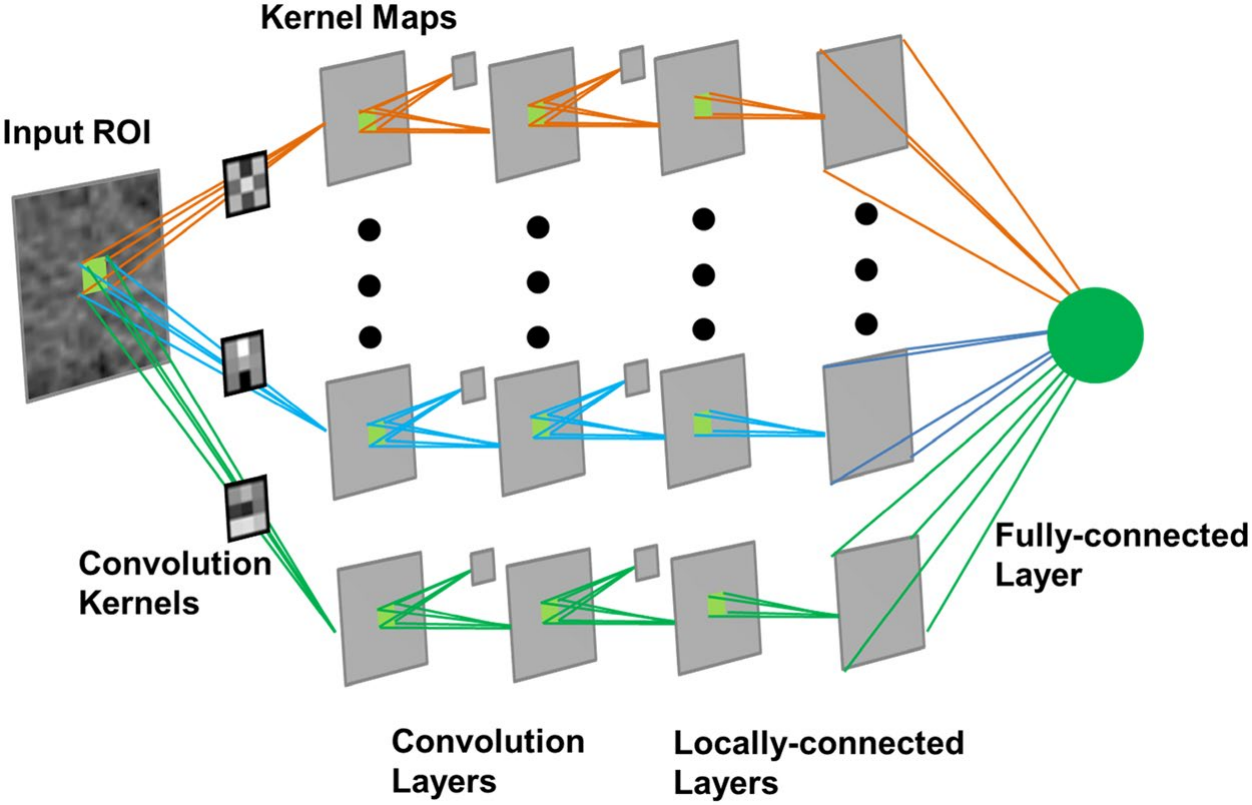
DL-CNN Structure. An input ROI is convolved with multiple convolution kernels, and the resulting values are collected into the corresponding kernel maps. This process repeats for several layers, giving the “deep” convolutional neural network. The network used in this study contains two convolution layers and two locally-connected layers, each of which contains 16 kernels.

Bladder lesions detected on the CT images were segmented using our Auto-Initialized Cascaded Level Sets (AI-CALS) system^27^. The AI-CALS system uses preprocessing, initial segmentation, and level set segmentation to differentiate bladder lesions from surrounding tissue and the bladder lumen. Figure 1 shows examples of segmented lesions. Regions of interest (ROIs) were extracted from within the segmented lesions from corresponding pre- and post-treatment scans of a patient and were paired together in multiple combinations to generate pre-post-treatment paired ROIs (Fig. 2). A total of 6,700 pre-post-treatment paired ROIs were generated (Fig. 3). We trained a DL-CNN to distinguish between bladder lesions that were diagnosed as stage T0 post-treatment (no residual tumor) and those that were greater than stage T0 (any residual tumor) (Fig. 4).

### RF-SL Predictive model

For this predictive model, a radiomics-feature-based analysis was applied to the segmented lesions (RF-SL). Multiple features were extracted from the segmented lesions identified on the pre and post-treatment CT images, including shape, size, and texture characteristics. Feature selection was performed, and a random forest classifier was trained to use these features to distinguish between lesions that fully responded to treatment and those that did not.

### RF-ROI Predictive model

In this model, the radiomics-feature-based analysis was applied to the paired ROIs (RF-ROI). The gray-level features and texture features were extracted from the paired ROIs. Similar to the RF-SL model, feature selection was performed, and a random forest classifier was trained to use these features to distinguish between lesions that fully responded to treatment and those that did not.

### Expert physician performance

An observer study with two experienced fellowship-trained abdominal radiologists, one with 17 years of experience and the other with 27 years of experience, was performed as references for comparison with the three predictive models. Each radiologist independently read the pre- and post-treatment images of patients that were loaded side-by-side, and estimated the likelihood of the patient having stage T0 cancer post-treatment. The images were presented in a randomized order to reduce potential observer bias.

## Results

**Figure 5:**
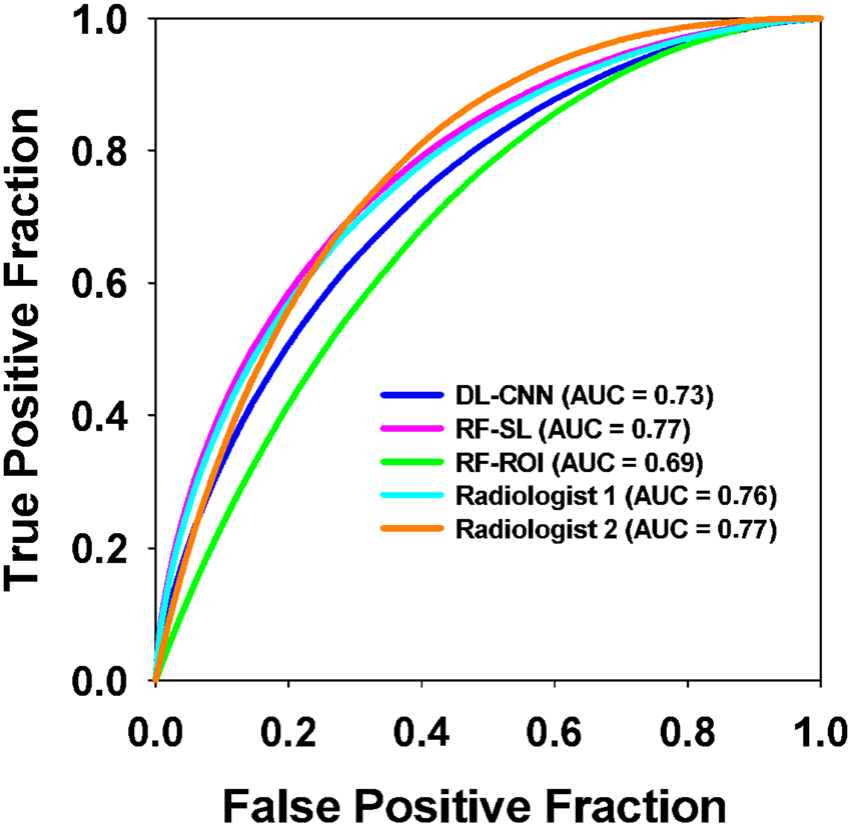
Test set ROC curves for the three models and two expert radiologists. The results from the test set for prediction of T0 stage after neoadjuvant chemotherapy for the three models. The differences between any pairs of AUCs did not reach statistical significance.

Receiver operating characteristic (ROC) analysis was performed and the area under the curve (AUC) was calculated as a measure of performance. Figure 5 shows the ROC curves for the DL-CNN, RF-SL, and RF-ROI methods and the radiologists for the test set. Table 1 shows the performances for the DL-CNN, RF-SL, and RF-ROI methods, along with the radiologists’ results for the test set.

**Table 1:** Performances of bladder cancer treatment response assessment in the test set.

	DL-CNN	RF-SL	RF-ROI	Radiologist 1	Radiologist 2
**AUC**	0.73 ± 0.08	0.77 ± 0.08	0.69 ± 0.08	0.76 ± 0.08	0.77 ± 0.07

DL-CNN: Deep-learning convolution neural network. RF-SL: Radiomics features extracted from segmented lesions. RF-ROI: Radiomics features extracted from pre- and post-treatment paired ROIs. The area under the curve (AUC) is shown with the standard deviations.

**Figure 6:**
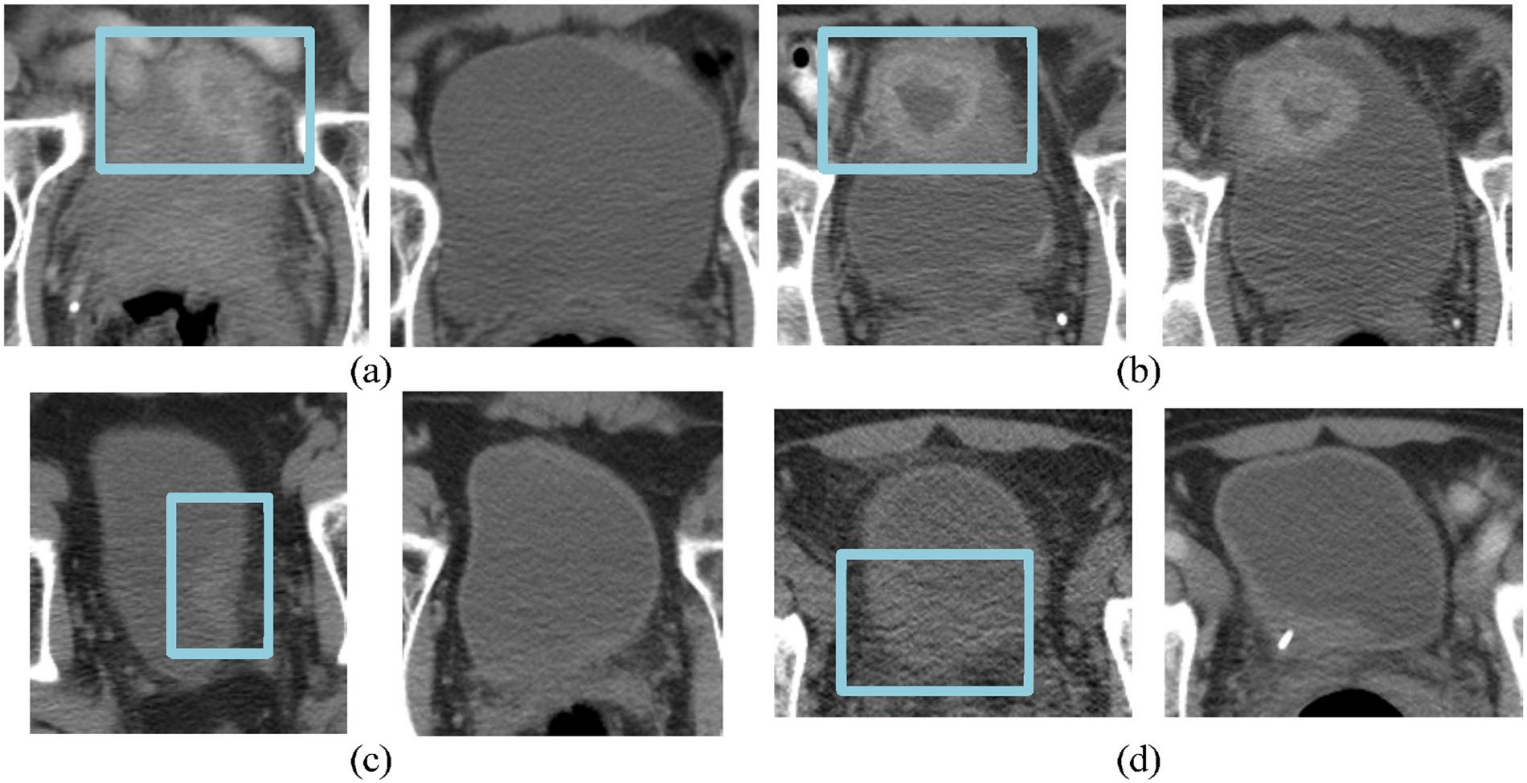
Examples of pre- and post-treatment bladders and their predictions. (**a**) The computer methods and the radiologists correctly predicted the treatment outcome for this case, which was a non-responding, progressive disease that went from stage T2 before treatment to T3a after treatment. (**b**) In this stable disease case (stage T3), the computer methods and the radiologists correctly identified the case as non-responding. (**c**) This case fully responded, going from stage T2 to T0, and the computer methods and the radiologists correctly predicted the treatment response. (**d**) A full-responding case, going from stage T3 to T0. The computers correctly predicted the response, while the radiologists did not. The region around the right ureterovesicular junction was asymmetrically thickened, which might have misled the radiologist to assess that cancer was present. The pre-treatment scan is on the left and the post-treatment scan is located on the right of each pair. The box on the pre-treatment scan represents the location of the lesion as marked by one of the radiologists.

The areas under the curve (AUC) for prediction of T0 disease after treatment were similar. The performances of all three methods are comparable to those of the radiologists. The differences between any two AUCs did not reach statistical significance. Examples of the treatment response prediction of pre- and post-treatment case pairs are shown in Fig. 6.

## Discussion

In this study, we evaluated a system that can distinguish between bladder cancers that have completely responded to neoadjuvant chemotherapy from those that have not, based upon computer analysis of pre- and post-treatment CT images. Although we used CT images before and after completion of three cycles of chemotherapy, we expect that the trained predictive models will be applicable to any treatment time points because they are trained to recognize the change in the image patterns to T0 stage regardless of when it occurs. If the models are validated, the models may be used to assess treatment response at any clinically relevant time point, and the treatment may be stopped or changed prior to the appearance of other toxic effects if the cancer is resistant to the treatment. The performance of the DL-CNN was compared against a radiomics feature-based method, where the percent change in the features extracted from the segmented lesions pre- and post-treatment was used (RF-SL), and against a third method extracting radiomics features from the paired ROIs used by the DL-CNN (RF-ROI).

For the RF-SL, five features were consistently selected which included a contrast feature and four run length statistics texture features. We have also performed an ordering of importance on the radiomics features based on the AUC of the individual features, and found that the selected features were highly ranked. For the RF-ROI, the gray-level average, the skewness of the gray-level histogram, and two run length statistics texture features were consistently selected. These results show that the texture, which characterizes the heterogeneity, of the bladder lesions is an important indicator for the estimation of full responders to chemotherapy. Detailed description of the selected features can be found in Supplementary Appendix B.

All three methods performed comparably in terms of AUC to the two expert radiologists. The RF-SL performed slightly better than the DL-CNN; however, the RF-ROI method resulted in worse performance compared to the DL-CNN, indicating that the DL-CNN is able to better characterize the paired ROIs to identify full responders compared to extracting features from the ROIs and using the random forest classifier. The absence of contrast material in some of the CT scans did not affect the results of the computer system or the radiologists. The pre- or post-treatment cancer stage did not have an observable effect on the performances of the system or the radiologists either.

The ROC curve provides a tradeoff in the sensitivity (true positive fraction) and specificity (true negative fraction) for predicting one class versus the other over the entire range of decision threshold (operating point). If the user of a decision support system prefers a binary recommendation (i.e., whether a case is predicted to be class 1 or class 2), a decision threshold for stratifying the two classes will need to be selected based on the training set and the system will output a class label for an unknown case being assessed. Table 2 shows the number of correctly identified cases in the complete response and non-complete response classes at a selected operating point for each classifier. The results show that the sensitivity of correctly identified completely responding lesion pairs for the DL-CNN method and the RF-SL method are lower than the RF-ROI method and the two radiologists, whereas the specificity of correctly identifying the non-completely responding lesions for these two methods are higher than the RF-ROI method and the two radiologists. This example demonstrates the tradeoff in the correct classification of the two classes among the different methods at a selected operating point. The method which is the better choice for a given task depends on the clinical needs. For a given method, the sensitivity-specificity performance can be changed by adjusting the operating point along the ROC curve based on the clinical needs. The operating point in the example shown was selected to attain a certain sensitivity level for all three methods based on the training set. That the performance level at the selected operating point on the test set did not closely follow that on the training set was likely caused by our small data set such that the distributions of the training set and test set were not statistically similar. In general, when a large enough data set that is similar to the patient population is available for training, the trained classifier (or decision support system) will be more robust and the performance measures (sensitivity, specificity, AUC) and the operating point will be more generalizable to the population^28, 29^. We will continue to collect cases and improve the trained classifiers in future studies.

**Table 2:** Number of correctly predicted bladder cancer treatment response assessment of the test set at an operating point determined using the training set.

	DL-CNN	RF-SL	RF-ROI	Radiologist 1	Radiologist 2
Complete Response (Sensitivity)	6/12 (50%)	6/12 (50%)	8/12 (66.7%)	11/12 (91.7%)	11/12 (91.7%)
Non-complete Response (Specificity)	34/42 (81.0%)	33/42 (78.6%)	23/42 (54.8%)	18/42 (42.9%)	16/42 (38.1%)

DL-CNN: Deep-learning convolution neural network. RF-SL: Radiomics features extracted from segmented lesions. RF-ROI: Radiomics features extracted from pre- and post-treatment paired ROIs.

**Figure 7:**
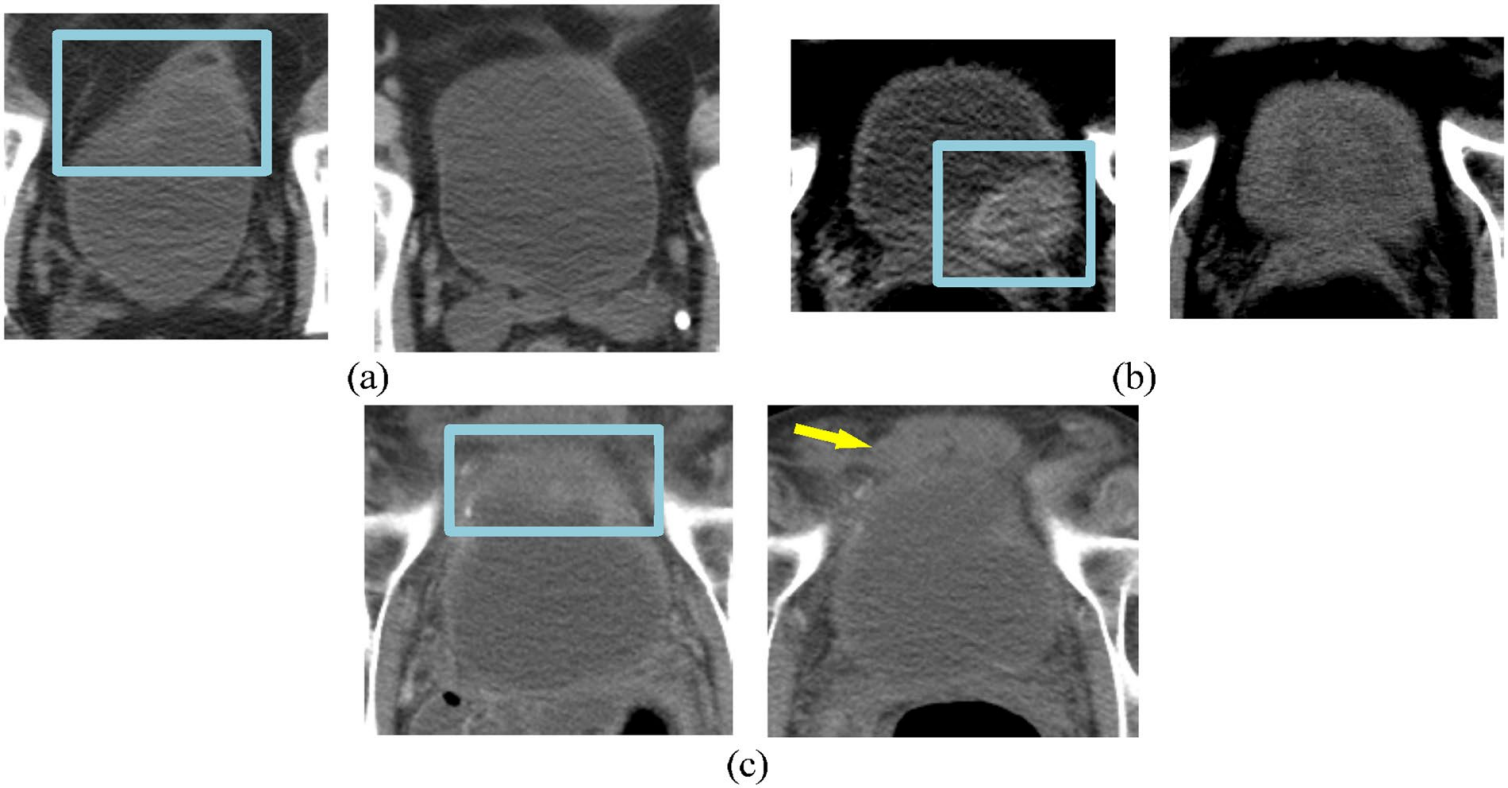
Examples of pre- (on the left side of each image pair) and post- (on the right side of each image pair) treatment bladders that responded fully to treatment, and the differences in the predictions by the computer models and radiologists. (**a**) All three computer methods and the radiologists correctly predicted the outcome of treatment for this case. (**b**) The three computer methods correctly identified the case as becoming T0 tumor, while the radiologists did not. There was residual bladder wall thickening, presumably related to the treatment, causing the radiologists to falsely conclude that there was persistent tumor. (**c**) The radiologist correctly identified that there was no residual tumor on post-treatment images, while the three computer methods failed to classify this case correctly. This was likely due to misidentification of perivesical tissue (arrow) as residual tumor by the computer models. The box on the pre-treatment scan represents the location of the lesion as marked by one of the radiologists.

Although the overall performance was similar across the methods, there are variations in the prediction performance in individual cases. Figure 7 shows examples of cancers that responded fully to treatment and the differences in the predictions by the computer models and the radiologists. In the case shown in Fig. 7(a), all methods and radiologists correctly identified that the detected cancer had responded fully to treatment. However, in the case shown in Fig. 7(b), the computer methods correctly identified the cancer as having a complete response, while the radiologists did not. Upon further review, the radiologists commented that the uncertainty was due to the presence of residual/persistent bladder wall thickening in the region of the tumor (which turned out to be benign at surgery). In the case shown in Fig. 7(c), the radiologists correctly identified the case as having responded fully to treatment, whereas the computer indicated that the case had not. The lesion in this case was located at the top of the bladder, and the automated computer segmented region incorrectly extended into the perivesical tissue outside the bladder. The analysis of this extravesical tissue by the computer models might have caused the incorrect decision that residual tumor was present.

**Figure 8:**
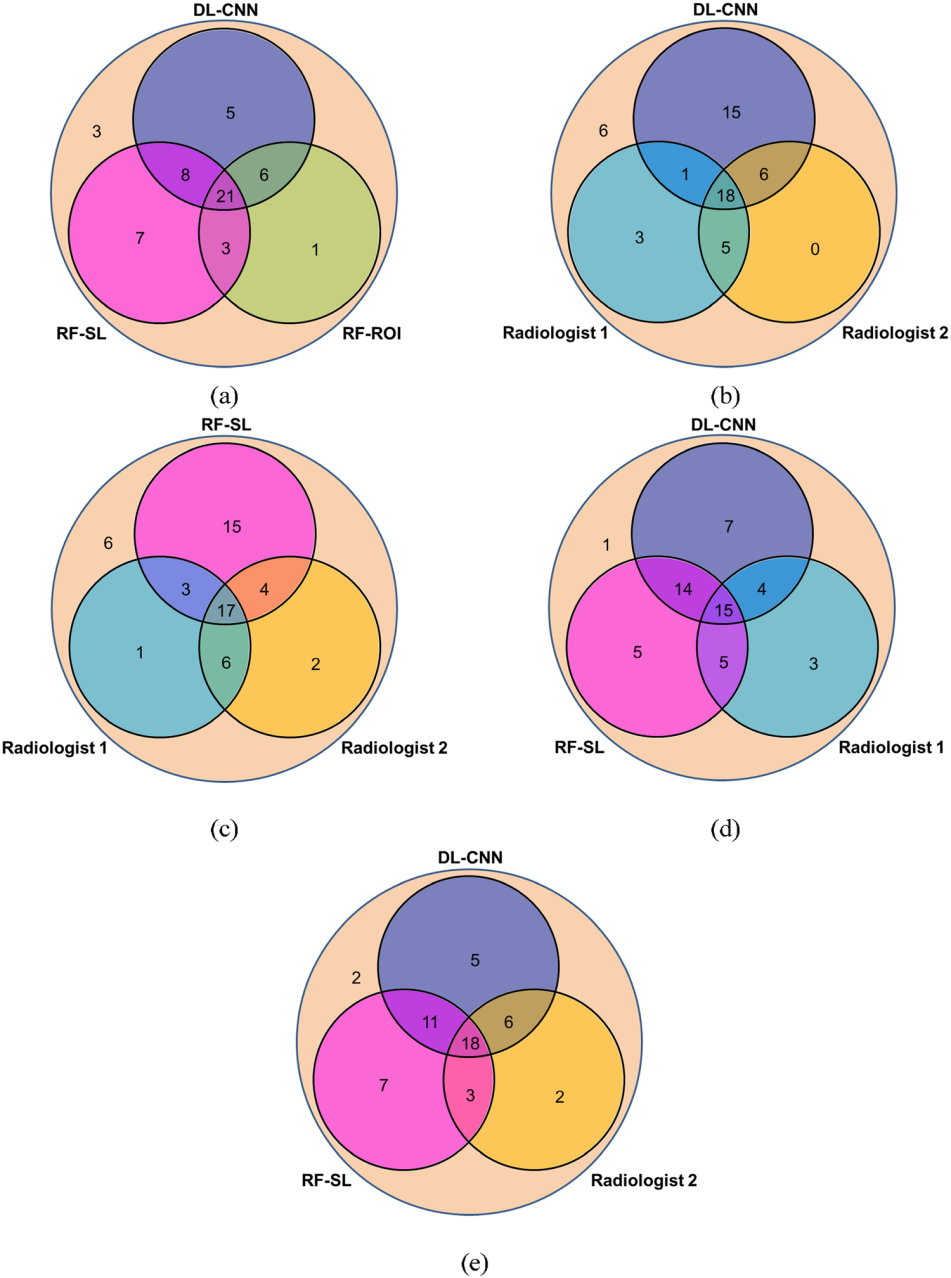
Venn diagrams of different methods and their assessments for the test set. The inner three circles compare the methods when at least one method correctly predicted the treatment outcome for a pre- and post-treatment pair. The outer circle contains the pre- and post-treatment pairs for which all three methods incorrectly predicted the treatment outcome. (**a**) The three computer methods correctly predicted the same outcome of the patients for 39% (21/54) of the pre- and post-treatment pairs. (**b**,**c**) The two radiologists correctly agreed on the outcome of 43% of the cases ((18 + 5)/54 for (**b**) and (17 + 6)/54 for (**c**)). (**d**,**e**) Radiologists 1 correctly agreed with the DL-CNN and the RF-SL methods for 19 and 20 cases, respectively, while Radiologist 2 correctly agreed with the DL-CNN and the RF-SL methods for 24 and 21 cases, respectively.

Given the fact that in some instances the computer models were correct about complete tumor responses and the radiologists were incorrect, we speculate that use of one or more of these models alongside a radiologist might improve the radiologist’s ability to identify patients who responds fully to chemotherapy. Figure 8 shows the Venn diagrams depicting the agreements of the assessments performed by the radiologists and the computer methods using the same operating point as that used for Table 2. In cases like that in Fig. 6(d), radiologists will generally decide that the case is a non-responder because they see residual bladder wall thickening, which is an indicator of cancer. If the computer models suggested that there was a high likelihood of T0 after treatment in this case, it might lead the radiologists to re-evaluate their decision, and, possibly come to a different (and correct) conclusion. In the case shown in Fig. 1(a), the tumor stage was found to be T2 at the time of subsequent cystectomy. Even though the computer indicated that the case had a high likelihood of having fully responded, the radiologist might have concluded that cancer was still present on the post-treatment images and decided not to be influenced by the computer’s opposite (and incorrect) prediction. Such decisions could lead to a higher accuracy in determining whether a patient had responded completely to neoadjuvant chemotherapy. Of course, it is also possible for the computer models to sway a radiologist’s decision in the wrong direction.

All currently approved Food and Drug Administration (FDA) computer-aided detection (CAD) devices are labeled for use as “second readers”, i.e., the workflow is to use the CAD system as a second opinion rather than a first reader or concurrent reader. For future decision support systems for treatment response assessment or other applications, it is unknown what the best approach is at this point. One possibility is to follow the second opinion approach: the clinician will first make his/her assessment without being influenced by the computer. The computer then shows the clinician its prediction. The clinician may reconsider his/her assessment using the computer’s prediction as additional information. Alternatively, a concurrent approach could be used, where prediction by the decision support system may be displayed from the beginning and it is up to the clinician to use the prediction as additional information in his/her decision making process. Generally, when clinicians’ assessments differ from each other, they may discuss and try to reach consensus or bring in additional expert opinion. If the clinician disagrees with the computer, he/she can override it because the computer models, in the current state of development, does not consider many other factors, such as clinical, genetic, or demographic factors, which a clinician may figure into their decision. The clinician may also bring in additional expert opinion if he/she deems it necessary.

Further study of the accuracy of the computer models in tandem with radiologist assessment is needed to determine whether or not such decision support systems will improve radiologist performance in treatment response assessments for bladder cancers. Previous observer performance studies showed that when radiologists and the computer systems had comparable performances individually, using the computer system to assist the radiologists improved their performance for breast cancer detection^30^, breast mass classification^31, 32^, and colon polyp detection^33^. We plan to perform an observer performance study using either the second opinion approach or the concurrent approach to evaluate if a similar trend will be observed for bladder cancer treatment response assessment.

There are several limitations to this study. First, the results of this study need to be improved. An AUC values in the range of 0.69 to 0.77 is not optimal for distinguishing between fully and partially or non-responding bladder cancers. Nevertheless, our feasibility study is the first to demonstrate the promise of using radiomics methods, as well as the DL-CNN to distinguish patients who respond to bladder cancer treatment from those who do not. Second, our pilot training data set was relatively small, consisting of only 82 patients with bladder cancer and subsequent generation of 6,700 ROIs. This is a very small data set for all classifiers in general and a small number of ROIs for training a DL-CNN in particular. The limited training set size could be a major factor that impacted the performance of a DL-CNN, compared to other pattern recognition tasks such as natural scene classification that can collect millions of training samples much more easily than collecting medical images. This pilot study indicates the potential of using DL-CNN and radiomics methods for treatment response prediction. We still need to study in greater detail the generalizability of the methods using larger data sets in future studies. A third limitation is that this study is a retrospective study. In the future, after the development is completed and the performance of the system is further improved, a prospective clinical trial should be conducted to assess its robustness in the population.

In summary, our study indicates that the computerized assessment using radiomics information from the pre- and post-treatment CT of patients who have undergone neoadjuvant chemotherapy for bladder cancer has the potential to assist in assessment of treatment response.

## Methods

### Lesion Segmentation

The AI-CALS system used for the segmentation of the bladder lesions consists of three stages: preprocessing, initial segmentation, and level set segmentation. In the first stage, preprocessing techniques are applied to a user-defined 3D region surrounding the bladder lesion. Smoothing, anisotropic diffusion, gradient filters and the rank transform of the gradient magnitude are applied to the slices within the region to obtain a set of smoothed images, a set of gradient magnitude images, and a set of gradient vector images. The set of smoothed images is used in the second stage, while the other two sets are used during level set propagation in the third stage. In the second stage, an initial segmentation surface is generated by thresholding the defined region in the smoothed images. Voxels in the 3D region that are within 3 standard deviations of the average gray level are labeled as the object region. A morphological dilation filter with a spherical structuring element of 2 voxels in radius, 3D flood fill algorithm, and a morphological erosion filter with a spherical structuring element of 2 voxels in radius are applied to the object region to connect neighboring components and extract an initial segmentation surface. In the third stage, the initial segmentation surface is propagated towards the lesion boundary using cascading 3D and 2D level sets. Additional information regarding the AI-CALS system can be found in literature^27^.

### ROI Creation

To train the DL-CNN with both the pre- and post-treatment bladder lesion information, we generated a single image containing information from the bladder lesion at the two time points. From every slice of a segmented bladder lesion, ROIs with dimension 16 × 32 pixels that were shifted with respect to one another but stayed within the segmented bladder lesion were extracted. If the bladder lesion was smaller than the ROI size, a single ROI centered at the centroid of the bladder lesion was extracted. Once these ROIs were extracted from both the pre- and post-treatment CT of a temporal lesion pair, the ROIs were combined by pasting them side-by-side, with a pre-treatment lesion ROI located on the left half and a post-treatment lesion ROI located on the right half of the combined ROI. The resulting image was a paired 32 × 32-pixel ROI containing both pre-and post-treatment information (see Fig. 2). Different combinations of the pre- and post-treatment lesion ROIs from the same case were used to form multiple paired ROIs. Each paired ROI was labeled as having or not having responded completely to treatment based on whether the cancer went down to stage T0 or not after treatment as determined by clinical information from the patient files. If the post-treatment ROI of a paired ROI was stage T0, the paired ROI was labeled as having complete response, but if the stage was greater than T0, the paired ROI was labeled as not having complete response. Figure 2 shows an example of how a paired ROI was formed and examples of paired ROIs and their labels.

### DL-CNN

A DL-CNN learns patterns of different classes from a set of labeled training images that are representative of the population of patterns being classified. After the user specifies a few parameters that control the training process, the DL-CNN reads the labeled images and learns the differences between the classes without any additional inputs. We applied the DL-CNN developed by Krizhevski *et al*., called cuda-convnet^34, 35^, to the classification of the paired ROIs with complete response, and those without. The parameters of the original network were modified, and a set of parameters was found that worked best for the task of identifying cases that completely responded to treatment using the training set. The network consists of five main layers: two convolution layers, two locally-connected layers, and one fully-connected layer. The locally-connected layers perform the same operation as the convolution layer except that, instead of applying a single convolution kernel to every location of the input image to obtain a kernel map, different convolution kernels are applied to every location of the input image and the resulting values are collected into the corresponding neurons within the corresponding kernel map. The fully-connected layer uses every kernel map element multiplied by a weight as input. Pooling and normalization are performed after each of the two convolution layers. All convolution layers consist of 16 kernels with a kernel filter size of 3 × 3. The parameters used for the DL-CNN can be found in Supplementary Appendix A. The “per-lesion” score was obtained by using the average value among the ROIs associated with the lesion.

### Radiomics Features from Segmented Lesions (RF-SL)

The conventional method for radiomics is to extract appropriate features from the segmented objects and characterize the objects with the features. We extracted 91 radiomics features from every segmented lesion. Four types of features were extracted: (1) morphological features, such as volume, circularity, rectangularity, and Fourier descriptor, (2) gray level features, such as the average gray level and contrast features, (3) texture features, such as run length statistics, and (4) gradient field features, such as the gradient magnitudes statistics for all voxels on the surface of the segmented lesion. Shape-based features and the contrast features were extracted from the central slice of the lesion. Other features, such as the gray-level features, texture features, and the gradient field features, were extracted from the segmented lesion in 3D after performing interpolation to obtain isotropic images. For every temporal lesion pair, the percent change between each radiomics feature extracted from the pre- and post-treatment lesion was calculated. These features were found to be potentially useful for lesion classification from our previous experience with breast masses and lung nodules^36, 37^. Features that were useful specifically for the bladder cancer treatment response assessment were further identified during the training of the predictive model. The percent change of each of the feature values before and after the treatment was calculated. Feature selection was performed and a random forest classifier using 6 trees and the minimum number of observations per tree leaf set of 13 was trained to use the selected radiomics features to predict the likelihood of the post-treatment lesion being T0 stage. The parameters were selected experimentally using the training set.

### Radiomics Features from Paired ROIs (RF-ROI)

Gray-level and texture features were extracted from the paired ROIs used for the DL-CNN. Thirty-eight features, including gray-level histogram statistics, and run length statistics features, were calculated for every ROI. The “per-lesion” features were generated by averaging the feature values among the ROIs associated with the lesion. Similar to the RF-SL model, feature selection was performed and a random forest classifier using 2 trees and the minimum number of observations per tree leaf set of 29 was trained to use the selected radiomics features to predict the likelihood of the post-treatment lesion being T0 stage. The parameters were selected experimentally using the training set.

### Performance Evaluation

For the three methods, the trained model was applied to the test set and the area under the curve (AUC) was calculated using the scores from the test cases. For the radiologists, the AUC was calculated for the test cases using their given likelihood of complete response to treatment.

### Data Set

Our study is a retrospective study. The CT scans of patients used in this study were acquired for their clinical care, no additional scans were acquired for the purpose of this study, the results did not influence the clinical care of the subjects in this study in any way, and all the patient data were de-identified before being used. Patient informed consent was waived by the IRB for this retrospective study so that informed consent was not obtained from the individual subjects. The patient images were collected and de-identified using methods approved by our Institutional Review Board (IRB) and are HIPAA compliant. All methods were performed in accordance to the guidelines and regulations of the IRB. A training data set of 82 patients (67 males, 15 females, age 64.0 ± 10.6, age range 37–84) with 87 bladder cancers was collected. The CT scans used in this study were acquired in our clinic with GE Healthcare LightSpeed MDCT scanners, acquired using 120 kVp and 120–280 mA. A total of 172 CT scans (pixel size range 0.586–0.977 mm, slice thickness range 0.625–7 mm) were obtained for the training set, of which 28 scans were performed without contrast material, while the remaining 144 scans were contrast-enhanced CT scans. Using the 87 lesions, 104 temporal lesion pairs were generated. Twenty-seven percent (28/104) of the lesions pairs had T0 cancer after neoadjuvant chemotherapy, which corresponds to a complete response to treatment, using the clinical information available from the patient files as reference truth. These temporal lesion pairs were used to generate 6,700 pre-post-treatment paired ROIs for use with DL-CNN and RF-ROI.

In addition, a test data set of 41 patients (33 males, 8 females, age 60.9 ± 9.2, age range 42–84) with 42 lesions was collected. A total of 88 CT scans (pixel size range 0.586–0.977 mm, slice thickness range 0.5–7.5 mm) were obtained for the test set, of which 16 scans were performed without contrast material, while the remaining 72 scans were contrast-enhanced CT scans. Fifty-four temporal pairs were generated from the 42 lesions. Twenty-two percent (12/54) of the lesion pairs had T0 cancer after neoadjuvant chemotherapy.
